# Non-Neurological Complications after Mechanical Thrombectomy for Acute Ischemic Stroke: A Retrospective Single-Center Study

**DOI:** 10.1155/2022/5509081

**Published:** 2022-12-23

**Authors:** Pierre Goffin, Romain Thouny, Julien Guntz, Denis Brisbois, Philippe Desfontaines, Pierre Demaret

**Affiliations:** ^1^Department of Anesthesia and Intensive Care, MontLegia Hospital, Groupe Santé CHC, Liège, Belgium; ^2^Faculty of Medicine, University of Barcelona, Barcelona, Spain; ^3^Department of Radiology, MontLegia Hospital, Groupe Santé CHC, Liège, Belgium; ^4^Department of Neurology, MontLegia Hospital, Groupe Santé CHC, Liège, Belgium

## Abstract

**Introduction:**

The global burden of stroke is high and mechanical thrombectomy is the cornerstone of the treatment. Incidences of acute non-neurological-complications are poorly described. Improve knowledge about these complications may allow to better prevent, detect and/or manage them. The aim is to identify risk markers of death or poor evolution.

**Method:**

We conducted a retrospective single-center study to analyzed the incidence of non-neurologicalcomplications after mechanical thrombectomy in acute ischemic stroke. Patients who had experienced a stroke and undergone thrombectomy were identified using a registry in which we prospectively collected data from each patient admitted to our hospital with a diagnosis of stroke. Quantitative and qualitative variables were analyses. The association between studied variables and hospital death was assessed using simple logistic regression models.

**Result:**

361 patients were reviewed but 16 were excluded due to a lack of medical information. Between 2012 and 2019, 345 patients were included. The median admission NIHSS score was 15. Seven percent of the patients died in the ICU. The following independent risk markers of death in the ICU were identified by logistic regression: respiratory complication, hypotension, infectious complication, and hyperglycemia.

**Conclusion:**

In this large retrospective study of stroke, respiratory complications and pulmonary infections represented the most important non-neurological adverse events encountered in the ICU and associated with a risk of death.

## 1. Introduction

Because of the growing and aging of the world's population, the global burden of stroke is high. Epidemiological data indicate that 16.9 million people experience a stroke each year worldwide, representing a global incidence of 258/100,000/year [1].

In 2015, endovascular mechanical thrombectomy (MT) aiming to remove the occluding clot in addition to intravenous thrombolysis was suggested in order to improve the recanalization rate. Indeed, the success rate of intravenous thrombolysis alone in patients with brain ischemia due to the occlusion of a major cerebral artery was deemed unsatisfactory [2]. Nowadays, the standard of care for emergency treatment of acute ischemic stroke (AIS) includes intravenous thrombolysis combined with mechanical thrombectomy which have been demonstrated to be associated with improved functional outcomes [3, 4]. Moreover, the expansion of the time window for reperfusion treatment with perfusion brain imaging screening has recently been found to be safe and effective [3]. Stroke remains one of the leading determinants of severe disability and death globally. Although endovascular therapy with mechanical thrombectomy (MT) is a formidable treatment for stroke management, a considerable proportion of patients do not experience clinical improvement, despite successful recanalization of the occluded artery and reperfusion of the ischemic region [5].

Neurological complications after MT for stroke have been largely described [6]; however, data on non-neurological complications developing in the intensive care unit (ICU) after stroke are scarce, including cardiovascular, respiratory, and infectious complications. However, considering the improved survival after critical illness, there is a focus on non-neurological complications that are of high importance since they may contribute to a reduced quality of life and a poor prognosis after a stroke [7]. Thus, it is important to gain more knowledge about these complications to better prevent, detect, and/or manage them.

To this end, considering the lack of published data on this topic, we conducted this retrospective single-center study to elucidate the non-neurological complications that occur during ICU stay after MT for ischemic stroke management.

## 2. Materials and Methods

### 2.1. Study Design, Study Site, and Population

**Figure 1:**
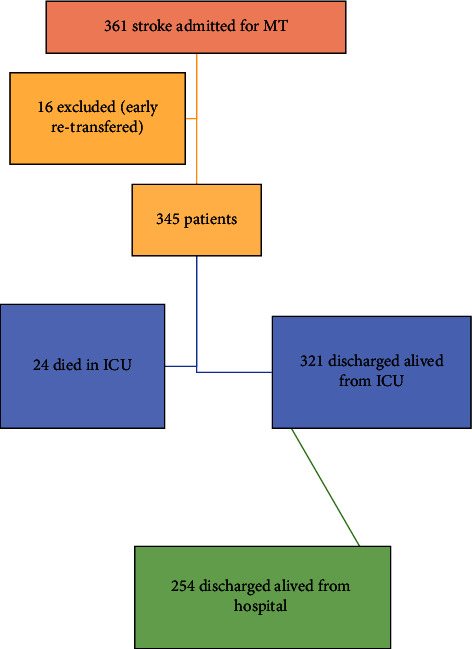
Patients flowchart.

This retrospective single-center study was conducted in Liège (Belgium). MT was implemented in our hospital in August 2011 [8]. Patients who had experienced a stroke and undergone MT were identified using a registry in which we prospectively collected data from each patient admitted to our hospital with a diagnosis of AIS. Patients admitted within 8 h after the onset of stroke, with proximal occlusion in the anterior cerebral region were considered. Patients who were treated at our hospital between August 1, 2012, and December 31, 2019, were included (Figure 1). Patients who were secondarily transferred to our center were also considered for screening. Those who were rapidly (within 48 h of ICU admission) retransferred to the referring hospital because of a lack of access to the data pertaining to their ICU and hospital stays were excluded.

Each patient admitted to our hospital with suspected or confirmed acute ischemic stroke (AIS) was first assessed in the emergency department (ED). General management was conducted by an emergency physician, while a neurological examination including determination of the National Institute of Health Stroke Scale (NIHSS) score was performed by a neurologist. If indicated, endovascular treatment was promptly conducted under general anesthesia (GA) by a neurointerventional radiologist. After MT, patients were systematically admitted to our 48-bed ICU for close monitoring. Computed tomography (CT) of the head was systematically performed 12 to 24 hours after MT or earlier in cases of neurological deterioration. After discharge from the ICU, patients were admitted to the stroke unit led by neurologists.

### 2.2. Data Collection

Data were retrospectively retrieved from the electronic medical records by two investigators. The baseline characteristics included age at ICU admission, sex, comorbidities, antiplatelet or anticoagulation therapy, and NIHSS score. Infectious complications were documented per medical notes. Stroke-associated pneumonia (SAP), pneumonia developing during the first 7 days after stroke onset in nonventilated patients, was recorded [8]. For cardiovascular complications, hyper- and hypotension were defined by the need for medication (hypotensive and vasoactive drugs, respectively) to maintain arterial blood pressure in the normal range [9], while new-onset arrhythmias were documented per medical notes and/or the need for an antiarrhythmic drug. A patient was considered to have a respiratory complication based on the need for invasive or noninvasive ventilation or an oxygen supply of ≥5 L/min. Hyperglycemia was defined as a blood glucose concentration of >180 mg/dL. Renal function was assessed using the Kidney Disease: improving Global Outcomes classification [10]. The pain was defined as a visual analog scale score of >3. Furthermore, the patients were monitored for complications related to the vascular access needed for MT, including local hematoma requiring intervention, vascular dissection, and limb ischemia. ICU and hospital survival data were also collected, and the NIHSS score was documented on days 2–4 and at the time of hospital discharge. [11] The study was approved by the ethics committee of the University of Liège, Belgium (reference number 2019-365). Patient consent was not required according to Belgian law and retrospective study protocol. Data were totally anonymized.

### 2.3. Statistical Analysis

The quantitative variables were expressed as medians and interquartile ranges (IQR). Qualitative variables were described using frequency tables (numbers and percent).

The association between studied variables and hospital death was assessed using simple logistic regression models. Variables for which the bivariate *p* value was less than 0.10 were introduced in a multiple logistic regression model and a backward stepwise selection method was applied to simplify the model.

The evolution over time of hemoglobin and renal function were analyzed using Student's *t*-test for paired samples. The results were considered statistically significant at the 5% level (*p* < 0.05). Analyzes were performed using SAS (version 9.4) and R (version 3.6.1) software.

## 3. Results

In all, 361 patients with AIS were treated with MT at our institution between August 1, 2012, and December 31, 2019. Sixteen patients were excluded because of early retransfer to the referring hospital (missing data), and finally, 345 patients were included for analysis. MT incidence in our institution has increased over the years, and 54.7% of the patients underwent MT in 2018–2019. The patients' demographic characteristics are shown in Table 1. The median patient age at hospital admission was 71 (61–80) years, and 53% of the patients were female. The median admission NIHSS score was 15 [10–18]. Fifty-five percent of the patients were treated within 4.5 h after stroke onset, 90% of whom underwent intravenous thrombolysis. Forty-five percent of the patients were treated between 4.5 and 8 h after stroke onset. Information about the MT procedure is presented in Table 2.

The median length of stay in the ICU was 3 [2, 3] days. Non-neurological complications during the ICU stay are shown in Table 3. Sixty-five (19%) patients presented with an infection, among which we documented 51 respiratory infections (global incidence of SAP: 14.8%) and 13 urinary tract infections; there was one case of meningitis. Almost 40% of the patients had arterial hyper- or hypotension. New-onset atrial fibrillation was observed in 69 patients (20%). Seventy-one patients (20.6%) had respiratory complications: 51 cases of SAP were documented, and 20 patients (5.8%) required mechanical ventilation because of hypoxemia and/or impaired consciousness. Hyperglycemia was noted in 51 patients (14.8%) during their ICU stay. Altered renal function occurred in three patients (0.8%). None of the patients required renal replacement therapy, and renal recovery was quick and complete. Pain (4.1%) and nausea (3.5%) were also observed in some patients. Other isolated complications are listed in Table 3.

Twenty-four patients (7%) died in the ICU. Three deaths were judged to be not directly related to neurological dysfunction and were linked to respiratory complications (respiratory sepsis, acute respiratory distress syndrome, and acute pulmonary edema). The remaining 21 deaths were directly attributable to AIS, including extended brain ischemia and/or intracranial hypertension. We found no correlation between the year of inclusion and the incidence of death (*p* = 0.79). The following independent risk factors of death in the ICU were identified by logistic regression analysis (Table 4): respiratory complications (adjusted odds ratio [OR]: 15, 95% confidence interval [CI]: 8–27, *p* < 0.001), hypotension (adjusted OR: 5.7, 95% CI: 2.8–12, *p* < 0.001), infectious complications (adjusted OR: 2.6, 95% CI: 1.5–4.6, *p* < 0.001), and hyperglycemia (adjusted OR: 4.9, 95% CI: 2.7–9.2, *p* < 0.001).

## 4. Discussion

In this study, we elucidated non-neurological complications that occurred after MT for stroke in proximal occlusion of the anterior cerebral circulation in a large population. Our group included patients with a median NIHSS score of 15 (severe stroke), which is a prognostic factor of poor outcome and may explain the high in-hospital mortality rate (33%). Half of our patients were treated in a late window, i.e., between 4.5 and 8 h after stroke onset, which might have led to an increase in the size of ischemic lesions. Nevertheless, the proportion (44, 81%) of good outcomes at 3 months (modified Rankin Scale score: 0–2) among the survivors was within the range reported in the literature.

Infections, such as pneumonia and urinary tract infections, greatly worsen the clinical outcomes of patients with stroke [12]. Infectious complications, particularly pulmonary infections, were frequent in our population. Pneumonia is a major complication of a stroke. Previously published work reported an incidence ranging from 4 to 56% [13]. Based on the application of standardized criteria, the occurrence of SAP was recently estimated to be 14%, which is in accordance with the incidence observed in our study [14]. A nasogastric tube, hemorrhagic conversion, and noninvasive ventilation have been described as risk factors for SAP [15]. Dysphagia and respiratory aspiration may partially explain the pathophysiology. In addition, stroke may induce complex immunodepression syndrome by disrupting the interplay between the central nervous and immune systems [12]. Respiratory complications is independent risk factors for in-hospital mortality and could be a modifiable factor [16].

Arterial hypertension may be present in patients before the stroke and is an important risk factor [17]. However, it appears that in the ICU, intravenous treatment is required, possibly due to undertreated hypertension despite personalized treatment and/or due to decompensation of hypertension in the context of stroke. In our study, we did not observe an independent association between hypertension and mortality. However, it has been shown that higher systolic blood pressure (BP) during the 24 h following MT is associated with poor outcomes [18]. BP < 160/90 mmHg was associated with better outcomes than more permissive hypertension [5]. In summary, hypertension can be considered a cause and source of complications. Decreases in baseline BP and hypotension during interventions have been found to be detrimental [21]. Recently, systolic BP reduction in the first 24 h after successful reperfusion was found to be inversely associated with poor outcomes [22]. We did not analyze the subgroup characteristics of hypotensive patients. However, hypotension may be partially attributable to the postanesthesia context or to the sedation required for patients who remain intubated after MT.

New-onset arrhythmia was observed during the ICU stay, which is in accordance with findings in other large cohorts [23, 24]. In a large US study, including more than 800.000 patients with AIS, acute myocardial infarction (AMI) was identified in 1.6% of cases, with the proportion of non-ST segment elevation myocardial infarction being 80% [25]. In our study, five patients (0.6%) were diagnosed with AMI, and two were discharged alive from the hospital. Hyperglycemia was identified as an independent risk factor for death. Stress hyperglycemia may also play a role in in-hospital outcomes [26]. Moreover, Mi et al. demonstrated a correlation between persistent hyperglycemia (glucose level: >140 mg/dL) within the first 24 h after stroke onset and patient mortality [27]. More recently, a high glucose level at admission (≥140 mg/dL) was associated with poor functional outcomes and an increased risk of symptomatic intracranial hemorrhage after endovascular treatment [20].

We attribute the decrease in hemoglobin level on the first day after stroke to per-procedure catheter and blood loss through catheter manipulation. This is probably also due to the intravenous dilution by fluid perfusion. In some cases, it may be related to a hematoma at the puncture site. In our experience, small hematomas at puncture sites are frequent. Fortunately, severe complications at the puncture site are rare; however, surgical treatment might be necessary, which might result in additional blood loss.

The early mortality rate after ischemic stroke is estimated to be approximately 15% in high-income countries [28]. We observed 7% of ICU mortality but 26% of in-hospital mortality. We did not analyze patient care projects that may have impacted the cause and/or timing of death. Indeed, acute stroke mortality may be more reflective of patient/family preferences than of the quality of care provided to hospitalized stroke patients. Decisions to withdraw or withhold interventions that might prevent or postpone mortality may considerably affect hospital-based mortality rates. Kelly et al. suggested that the timing of mortality after stroke may be affected by family preferences in more than 40% of the deaths. This suggests that death can occur despite the provision of high-quality care and reflects patient/family preferences regarding goals of care, especially in the elderly population [29]. In our center, MT is performed under GA. Although it is still debated, it has not been shown that GA for MT is associated with a worse outcome than conscious sedation [30]. A recent meta-analysis demonstrated that in centers with high-quality, specialized neuroanesthesia care, GA-treated thrombectomy patients had superior recanalization rates and better functional outcomes at 3 months compared with patients who received conscious sedation [31].

Our study has several limitations. First, owing to its retrospective design, there was a risk of information bias. Second, our data were obtained from a single center, which may have limited their external validity. Third, data after ICU discharge are scarce. Fourth, our inclusion timeline was quite long; hence, the results may have been affected by technical and care evolution, although 54.8% of our patients were included during the last 2 years of the study (2018–2019).

## 5. Conclusion

In this large study of patients with severe AIS, respiratory complications and pulmonary infections were the most important non-neurological adverse events encountered in the ICU and were independently associated with in-hospital mortality. Hypotension and hyperglycemia were identified as independent risk factors for death. Hypertension and arrhythmia were also frequent but were not associated with mortality. Awareness of potential complications after stroke treatment with MT may lead to better prevention and may ultimately lead to a better outcome.

## Figures and Tables

**Table 1:** Patients characteristics (*N* = 345).

Age (years)^*∗*^	71 (61–80)
Gender	
Female	183 (53.0%)
Male	162 (47.0%)
Hospital origin	
Other hospital	147 (42.6%)
Emergency department	198 (57.4%)
Comorbidity	
HTA	254 (73.6%)
Diabetes	60 (17.4%)
Thrombo-embolism disease	34 (9.9%)
Ischemic stroke	45 (13.0%)
Cardiovascular disease	103 (29.9%)
Respiratory disease	29 (8.4%)
Atrial fibrillation	80 (23.2%)
Oncologic history	25 (7.2%)
Postpartum	5 (1.4%)
Renal disease	21 (6.1%)
Obesity	64 (18.6%)
Active smoking	120 (34.8%)
Medication	
Antiplatelet therapy	134 (38.8%)
Simple	118
Dual	16
Anticoagulation	61 (17.7%)
Neurological severity	
NIHS on admission	15 (11–19)
Good outcome at 3 months: mRS 0 to 2	44.81%

^*∗*^Continuous variables are described as median (interquartile range).

**Table 2:** MT procedures information

	*N* = 345 (%)
Procedure characteristics	
End procedure extubation	308 (89.3)
Vasopressive drug support	80 (23.2)
Closing device (ProGlide™)	307 (89.0)
Carotid stenting	86 (24.9)
TICI scale^*∗∗*^	
1	20 (5.8)
2a	40 (11.6)
2b	82 (23.8)
3	202 (58.6)
Timing procedure (in minutes)	
1^st^ symptom to admission^*∗*^	60 (140–170)
1^st^ symptoms to puncture^*∗*^	240 (180–360)

^*∗*^Continuous variables are described as median (interquartile range). ^*∗∗*^Thrombolysis in cerebral infarction scale.

**Table 3:** Non-neurological ICU complications.

System and type of complication		Number of patients (percentage) (#for continuous variables: median (interquartile range))
Pulmonary	Respiratory complication	71 (20.6)
	Acute pulmonary oedema	4 (1.2)
	Acute respiratory distress syndrome	1 (0.3)

Cardiovascular	Hemodynamic complication	
	Hypertension	103 (29.9)
	Hypotension	39 (11.3)
	Acute myocardial infarction	5 (1.4)
	NSTEMI	4 (1.2)
	STEMI	1 (0.3)
	Atrial fibrillation	69 (20.0)

Infectious	Infectious complication	65 (18.8)

Endocrine	Glycemia at *d* + 1 (mg/gL)	109 (99–140) #
	Hyperglycemia (≥180 mg/dL)	51 (14.8)

Hematological	Hemoglobin level (mg/dL)	
	At *d* + 0	13.4 (12.2–14.7) #^*∗*^
	At *d* + 1	12.2 (11.3–13.3) #^*∗*^

Renal	MDRD (mL/min)	
	At *d* + 0	75 (60–90) #^*∗∗*^
	At *d* + 1	78 (60–90) #^*∗∗*^
	Altered renal function (KDIGO 1–3)	3 (0.9)

Abdominal	Gastric perforation	1 (0.3)

Mechanical thrombectomy related	Femoral dissection = angioplasty and stenting	3 (0.9)
	Complicated hematoma = surgical treatment, one inferior limb ischemia, one inferior limb fasciotomy	4 (1.2)

Global	Pain (VAS > 3)	14 (4.1)
	Nausea/vomiting	12 (3.5)
	Death in ICU	24 (7.0)
	Death in-hospital (after ICU)	91 (26.4)

^*∗*^Difference 1.4 ± 1.3; *p* < 0.0001, ^*∗∗*^Difference 0.21 ± 11.9; *p* = 0.7.

**Table 4:** Independent risk markers of death.

	OR (95% CI)	*p* value
Respiratory complications	15 (8,27)	<0.001
Hypotension	5.7 (2.8, 12)	<0.001
Infectious complications	2.6 (1.5, 4.6)	<0.001
Hyperglycemia	4.9 (2.7, 9.2)	<0.001

## Data Availability

The data used to support the study are included in the paper.

## Abbreviations

ED: Emergency department
NIHSS: National institute of health stroke scale
ICU: Intensive care unit
T: Mechanical thrombectomy
AIS: Acute ischemic stroke
BP: Blood pressure
MI: Acute myocardial infarctions.
